# Percutaneous transforaminal endoscopic discectomy vs. unilateral biportal endoscopy for far lateral lumbar disc herniation: a retrospective comparative study

**DOI:** 10.3389/fsurg.2026.1790170

**Published:** 2026-04-22

**Authors:** Lu Yongjiang, Li Chunbo, Wang Jianyuan

**Affiliations:** Department of Orthopedics, Xinjiang 474 Hospital, Urumqi, Xinjiang, China

**Keywords:** comparative study, far lateral lumbar disc herniation, minimally invasive surgery, percutaneous transforaminal endoscopic discectomy, unilateral biportal endoscopy

## Abstract

**Objective:**

To compare the early clinical efficacy, perioperative parameters, and safety profiles of percutaneous transforaminal endoscopic discectomy (PTED) and unilateral biportal endoscopic (UBE) discectomy in treating single-level far lateral lumbar disc herniation (FLLDH).

**Methods:**

This retrospective cohort study analyzed 81 patients with FLLDH treated between January 2021 and June 2024. Patients were allocated to the PTED group (*n* = 38) or the UBE group (*n* = 43) based on the surgical technique received. Perioperative indicators (operative time, incision length, blood loss, hospital stay) and clinical outcomes—assessed by Visual Analogue Scale (VAS) for back/leg pain and the Oswestry Disability Index (ODI) preoperatively and at 1, 3, 6, and 12 months postoperatively—were compared. Statistical analyses included independent samples t-tests, chi-square tests, and repeated-measures ANOVA.

**Results:**

All patients completed 12-month follow-up. The two groups were comparable at baseline (*P* > 0.05). The PTED group demonstrated significantly shorter operative time (62.4 ± 8.7 vs. 105.3 ± 14.1 min, *P* < 0.001), smaller incision length (7.8 ± 0.9 vs. 24.6 ± 4.2 mm, *P* < 0.001), less intraoperative blood loss (18.5 ± 4.3 vs. 68.2 ± 10.5 mL, *P* < 0.001), and shorter hospital stay (4.8 ± 1.1 vs. 5.9 ± 1.7 days, *P* = 0.002). Both groups showed significant and sustained improvement in VAS and ODI scores postoperatively (*P* < 0.05). At 1 month, PTED was associated with lower back pain VAS but slightly higher leg pain VAS and ODI compared to UBE (*P* < 0.05). From 3 months onward, no significant inter-group differences were observed in any clinical scores (*P* > 0.05). Repeated-measures ANOVA indicated a different improvement trajectory for back pain between the groups (interaction *P* = 0.024).

**Conclusion:**

Both PTED and UBE are effective minimally invasive techniques for FLLDH, offering comparable and excellent mid-term clinical outcomes. PTED offers advantages in reduced surgical trauma and faster early recovery, whereas UBE provides superior endoscopic visualization. The choice of technique can be individualized based on patient characteristics and surgical expertise.

## 1 Introduction

Far lateral lumbar disc herniation (FLLDH) refers to a specific type of lumbar disc herniation in which the herniated material is located inside or outside the intervertebral foramen, directly compressing the exiting nerve root at the same segment. Since its first clear description by Abdullah et al. (1) in 1974, its reported incidence accounts for approximately 6.5%–12% of all lumbar disc herniations (2). Due to its unique anatomical location, FLLDH often leads to severe radicular leg pain and neurological dysfunction, significantly impacting patients' quality of life (3).

Traditional open surgical approaches, such as posterior laminectomy with facetectomy and foraminotomy, although effective in decompression, are often associated with extensive soft-tissue dissection and bony destruction, which may lead to complications such as postoperative lumbar instability and chronic low back pain (4). With the rapid advancement of minimally invasive spinal surgery concepts and techniques, percutaneous transforaminal endoscopic discectomy (PTED) has become a classic minimally invasive procedure for treating FLLDH (5). By operating through natural anatomical corridors or very small soft-tissue channels, PTED offers advantages such as minimal trauma and rapid recovery (6). In recent years, unilateral biportal endoscopic (UBE) technology has been rapidly adopted and applied in the treatment of lumbar spinal disorders due to its relatively gentle learning curve, spacious operative field, clear visualization, and compatibility with conventional open surgical instruments, providing another minimally invasive option for FLLDH (7).

Currently, both PTED and UBE are considered effective minimally invasive approaches for FLLDH (8, 9). However, the two techniques differ in surgical access, anesthesia method, operative logic, learning curve, and requirements for surgical experience (10). In clinical practice, determining the optimal choice between these two techniques based on individual patient characteristics, pathological features, and available medical resources still requires more robust evidence-based data (11). Although previous studies have separately reported the outcomes of each technique, systematic comparisons directly contrasting PTED and UBE in the treatment of FLLDH—particularly regarding short-term efficacy, perioperative indicators, and early recovery characteristics—remain insufficient (12).

Therefore, this study aims to conduct a retrospective cohort analysis to compare the early clinical outcomes, perioperative parameters, and safety profiles of PTED and UBE in the treatment of single-segment FLLDH. The study will analyze the respective advantages and limitations of both techniques, with the goal of providing evidence-based references for individualized selection of minimally invasive surgical approaches in clinical practice.

## 2 Materials and methods

### 2.1 General information

This study is a retrospective cohort analysis. Patients diagnosed with FLLDH and treated at our hospital between January 2021 and June 2024 were screened, of whom 81 met the predefined inclusion and exclusion criteria. According to the surgical technique received, patients were divided into two groups: 38 underwent percutaneous transforaminal endoscopic discectomy (PTED group), and 43 UBE discectomy (UBE group). The study protocol was reviewed and approved by the Ethics Committee of Xinjiang 474 Hospital. Due to the retrospective nature of the study, which involved no additional interventions and utilized only data from routine clinical records, the Ethics Committee granted a waiver of informed consent.

The selection of surgical technique followed a standardized clinical decision-making process: a multidisciplinary spine team individualized the choice based on anatomic factors assessed via preoperative imaging (e.g., foraminal morphology, iliac crest height, facet joint hypertrophy), herniation characteristics (size, calcification, migration degree), and the patient's preference regarding anesthesia. All patients provided fully informed consent before the final technique was determined, ensuring that group allocation was based on objective clinical indicators.

### 2.2 Inclusion criteria

(1) Presence of definite symptoms such as lower limb radicular pain, numbness, or muscle weakness consistent with the dermatomal distribution of the compressed nerve root; (2) MRI or CT confirmation of FLLDH (extraforaminal, intraforaminal, or mixed type) compressing the exiting nerve root at the corresponding level, with imaging findings correlating with clinical symptoms; (3) Failure of standardized conservative treatment or recurrence of symptoms for ≥6 weeks; (4) Undergoing PTED or UBE discectomy; (5) Completion of 12-month postoperative follow-up with complete pre- and postoperative imaging data available.

### 2.3 Exclusion criteria

(1) Multi-segment herniation accompanied by lumbar instability or spondylolisthesis; (2) Previous surgical history at the affected level; (3) Coexisting lumbar tumors, tuberculosis, active infection, or similar conditions; (4) Comorbid psychiatric, neurological, or cognitive disorders.

### 2.4 Surgical procedure

#### 2.4.1 Surgical team

All operations were performed by a single, fixed team of senior spinal surgeons who specialize in degenerative spinal conditions. Each surgeon had substantial experience and completed structured advanced training in both open and minimally invasive spinal surgery. With regard to the surgical techniques employed in this study, all surgeons were beyond the early learning curve for UBE and likewise proficient in PTED procedures, ensuring a consistently high level of technical skill and standardized operative execution across the entire cohort during the study period.

#### 2.4.2 PTED group

Taking a right-sided L₄_–_₅ FLLDH patient as an example. The patient was placed in the left lateral decubitus position with padding positioned at the lumbosacral junction to optimize surgical exposure. Under C-arm fluoroscopic guidance, the target intervertebral disc level was first identified, and a puncture target point was selected 8 cm lateral to the midline on the right side of the spinous process. After routine skin disinfection and draping, local infiltration anesthesia was administered along the planned puncture trajectory under imaging guidance, covering the facet joint area. Following anesthesia, an approximately 8 mm skin incision was made. A guidewire was inserted percutaneously and exchanged for a sequential dilation cannula system (4–8 mm) to establish the soft-tissue working channel. A trephine was used to partially remove bone from the superior articular process to enlarge the intervertebral foramen. Fluoroscopy confirmed that the trephine tip was positioned within the middle-outer third of the pedicle (Zone IV) on the anteroposterior view and directed toward the target herniated disc fragment on the lateral view. After placing the working cannula, the endoscopic imaging system was connected, and the procedure was performed under continuous saline irrigation. A bipolar radiofrequency probe was used for meticulous hemostasis during the operation. The working cannula was rotated to shield and protect the exiting nerve root. First, the herniated nucleus pulposus was removed from beneath the exiting nerve root (axilla) and sent for pathological examination. The cannula was then partially withdrawn to explore the dorsal and shoulder areas of the nerve root, where any hypertrophic tissue surrounding the nerve root was cleared and hemostasis achieved. The patient was instructed to elevate, internally rotate, and adduct the affected lower limb. Under endoscopic visualization, good nerve root mobility and vascular perfusion were observed, with ample space ventral, dorsal, cranial, and caudal to the nerve root, and no active bleeding within the spinal canal. Finally, the endoscopic system was withdrawn, and the incision was sutured and dressed.

#### 2.4.3 UBE group

Taking a left-sided L₄_–_₅ FLLDH patient as an example. Under general anesthesia, the patient was placed in the prone position with the abdomen free. The surgical area was routinely disinfected and draped. First, the responsible intervertebral disc level was localized using C-arm fluoroscopy. The incision point was marked approximately 4 cm lateral to the left spinous process (about 1 cm lateral to the outer margin of the pedicle projection, adjusted slightly according to patient body habitus). Two longitudinal incisions were made approximately 2–3 cm apart along the horizontal line of the L₄ superior articular process tip, measuring about 8 mm (observation portal) and 10 mm (working portal, ideally aligned with the L₄_–_₅ disc space) in length, respectively. After incising the deep fascia, the paraspinal soft-tissue channel was serially dilated using dilators centered on the L₄ pars interarticularis. An endoscopic cannula was inserted through the cranial portal, and a semi-open cannula device was placed through the caudal portal. Fluoroscopy confirmed the convergence of the semi-cannula instrument tip and the endoscope cannula tip at the lateral edge of the pars interarticularis. The endoscope and an endoscopic plasma electrode were then introduced. After managing the soft tissues, the lateral edge of the L₄ pars, the inferior border of the L₄ transverse process, and the L₅ superior articular process joint were clearly exposed. Using a high-speed burr and laminectomy rongeurs, partial bone was removed from the tip of the L₅ superior articular process, the lateral aspect of the L₄ pars, and part of the inferior border of the L₄ transverse process. After resecting the intertransverse ligament, the exiting nerve root (L₄ nerve root) of the same segment was exposed. Adhesions were released, and the herniated nucleus pulposus was removed and sent for pathological examination. Bipolar radiofrequency was used to repair the ruptured annulus fibrosus. A final inspection confirmed satisfactory nerve root pulsation, a patent neural foramen, and adequate decompression. Meticulous hemostasis was achieved under endoscopic visualization, followed by sequential closure of the incisions.

### 2.5 Postoperative treatment

Postoperative care included 24–48 h of prophylactic antibiotics, neuroprotective and analgesic medications, and early ambulation protocols. All patients received structured education on spinal biomechanics and ERAS-compliant pain control. For functional recovery, a soft lumbar brace was recommended for 4–6 weeks during ambulation only, with subsequent use discouraged to prevent core muscle deconditioning. Structured physical therapy—focusing on core strengthening and neuromuscular re-education—was initiated at 4–6 weeks postoperatively. Patients were advised to avoid heavy labor and high-impact sports for 3 months, with a gradual return to full activity thereafter based on individual functional recovery and radiographic evidence of fusion.

### 2.6 Observational indicators

1. Demographic and Baseline Clinical Data: Collected variables included age, gender, body mass index (BMI), surgical level, smoking history, alcohol consumption history, comorbidities (including but not limited to hypertension, diabetes, circulatory system diseases, neurological diseases, and respiratory system diseases), and follow-up duration.
2. Surgical Parameters: Parameters recorded comprised operative time, incision length, intraoperative blood loss (calculated using the fluid balance method: total drainage volume minus irrigation volume), and length of hospital stay. Clinical efficacy was assessed using the Visual Analogue Scale (VAS) scores for lower back pain and leg pain, and the Oswestry Disability Index (ODI).
3. Postoperative Imaging Evaluation: Postoperative lumbar dynamic x-rays, combined with three-dimensional CT reconstruction, were obtained to evaluate the extent of facet joint resection and assess lumbar spinal stability. Concurrent MRI examinations were performed to confirm the effectiveness of neural decompression and the status of soft tissue repair.
4. The facet joint resection ratio was calculated using pre- and postoperative parasagittal CT reconstructions with the following formula:
  Resection Ratio (%) = [1—(Postoperative Articular Process Height/Preoperative Articular Process Height)] × 100%. The articular process height was measured at the same anatomical landmark on CT images by two independent observers, and the average value was used for analysis.

### 2.7 Statistical analysis

Statistical analysis was performed using SPSS version 26.0 (IBM Corp.). Continuous variables with normal distribution are presented as mean ± standard deviation (x¯ ± s), and compared using independent samples *t*-tests. Categorical variables are expressed as numbers (percentages) and compared using chi-square or Fisher's exact tests. For repeated-measures data (VAS, ODI), repeated-measures ANOVA was applied to evaluate time and group effects and their interaction, with Greenhouse–Geisser correction if sphericity was violated. *Post-hoc* comparisons used Bonferroni adjustment. A two-sided *P* < 0.05 was considered significant. To address potential confounding in this observational design, we performed additional analytical adjustments. Propensity score matching (PSM) was conducted using 1:1 nearest-neighbor matching (calipe*r* = 0.2 SD) based on a logistic model including age, gender, BMI, surgical level, smoking, comorbidities, and herniation morphology (size, calcification, migration). Herniation size was defined as the maximum anteroposterior diameter (mm) measured on preoperative axial MRI and categorized as small (<5 mm), medium (5–10 mm), or large (>10 mm). Balance was assessed using standardized mean differences (SMD < 0.1 indicated good balance). Multivariable regression (linear for continuous outcomes, logistic for complications) was further used to adjust for age, BMI category, herniation type, migration grade, and surgical level. Adjusted results are reported as adjusted mean differences (aMD) or odds ratios (aOR) with 95% confidence intervals.

## 3 Results

### 3.1 The comparison of general data

**Figure 1:**
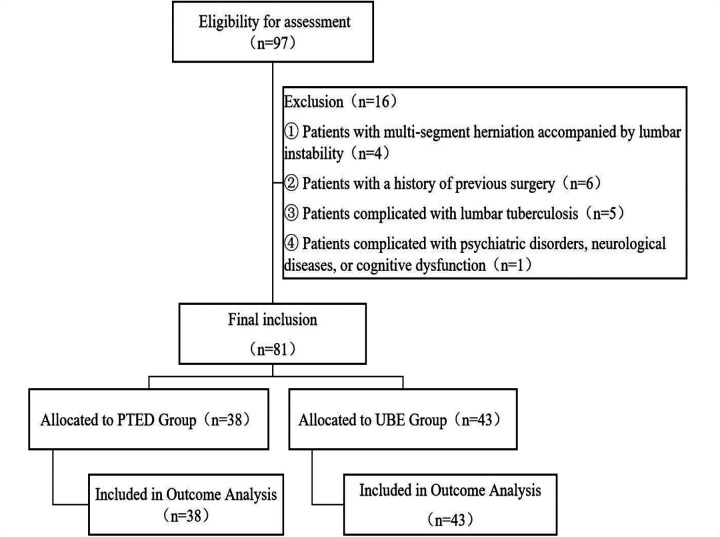
Flow chart of test grouping.

The study initially screened 97 patients with FLLDH. After applying the predefined exclusion criteria, 16 patients were excluded: 4 for multi-segment herniation with lumbar instability, 6 for previous spinal surgery, 5 for concomitant lumbar tuberculosis, and 1 for psychiatric, neurological, or cognitive disorders. Finally, 81 eligible patients were included and allocated to two surgical groups: 38 underwent PTED and 43 underwent UBE. All enrolled patients completed the 12-month follow-up and were included in the outcome analysis without any loss to follow-up (Figure 1). Baseline demographic and clinical characteristics were comparable between the two groups (*P* > 0.05), as detailed in Table 1.

**Table 1:** Comparison of the preoperative general data between the two groups.

Index	PTED group (*n* = 38)	UBE group (*n* = 43)	t/*χ*^2^	*P*
Age (year)	58.6 ± 10.2	57.1 ± 11.3	*t* = 0.647	0.520
Gender (n, Male/Female)	22/16	21/22	*χ*^2^ = 0.672	0.412
Body Mass Index (kg/m^2^,)	24.8 ± 3.1	25.2 ± 3.5	*t* = 0.548	0.585
Surgical Segment, n			*χ*^2^ = 1.824	0.610
L2/3	1	2		
L3/4	4	5		
L4/5	25	27		
L5/S1	8	9		
Smoking History, *n* (%)	12 (31.6)	15 (34.9)	*χ*^2^ = 0.103	0.749
Drinking History, *n* (%)	9 (23.7)	11 (25.6)	*χ*^2^ = 0.044	0.835
Comorbidities, *n* (%)				
Hypertension	14 (36.8)	17 (39.5)	*χ*^2^ = 0.066	0.798
Diabetes	8 (21.1)	7 (16.3)	*χ*^2^ = 0.302	0.583
Circulatory Disease	5 (13.2)	8 (18.6)	*χ*^2^ = 0.452	0.501
Neurological Disease	3 (7.9)	2 (4.7)	*χ*^2^ = 0.384	0.535
Respiratory Disease	4 (10.5)	3 (7.0)	*χ*^2^ = 0.333	0.564
Follow-up time (month)	13.1 ± 2.4	12.8 ± 2.1	*t* = 0.614	0.541

Values are given as the count or as the mean standard deviation (x̅ ± s). *χ*^2^:Chi-square test.

### 3.2 Comparison of surgical related indicators

All patients successfully completed the scheduled surgical procedure. In comparison to the UBE group, the PTED group demonstrated statistically significant reductions in several key perioperative metrics. Specifically, the PTED group had a substantially shorter operative time, smaller total incision length, less intraoperative blood loss, and a briefer postoperative hospital stay (all *P* < 0.05). See Table 2 for details.

**Table 2:** Comparison of intraoperative and early postoperative outcomes between the two groups.

Outcome indicator	PTED group (*n* = 38)	UBE group (*n* = 43)	*P*
Operative Time (min)	62.4 ± 8.7	105.3 ± 14.1	**<0.001**
Incision Length (mm)	7.8 ± 0.9	24.6 ± 4.2	**<0.001**
Intraoperative Blood Loss (mL)	18.5 ± 4.3	68.2 ± 10.5	**<0.001**
Length of Hospital Stay (days)	4.8 ± 1.1	5.9 ± 1.7	**0.002**

Values are given as the count or as the mean standard deviation (x̅ ± s). Bold numbers indicate that the two groups have statistical significance.

### 3.3 Propensity score matching and adjusted outcomes

To enhance the comparability of the two surgical groups, propensity score matching yielded 32 well-balanced pairs (PTED: *n* = 32, UBE: *n* = 32) from the original cohort. All baseline covariates, including herniation morphology, were balanced after matching (all SMDs < 0.1; see Table 3).

**Table 3:** Baseline characteristics of patients after propensity score matching.

Characteristic	PTED Group (*n* = 32)	UBE Group (*n* = 32)	SMD
Age, years	58.1 ± 9.8	57.9 ± 10.5	0.02
Gender, Male (%)	20 (62.5)	19 (59.4)	0.06
BMI, kg/m^2^	24.9 ± 3.0	25.1 ± 3.3	0.06
Surgical Level, L4/5 (%)	21 (65.6)	22 (68.8)	0.07
Calcified Herniation, *n* (%)	5 (15.6)	4 (12.5)	0.09
Migrated Herniation, *n* (%)	9 (28.1)	10 (31.3)	0.07
BMI >30, *n* (%)	4 (12.5)	5 (15.6)	0.09

SMD, standardized mean difference; SMD <0.1 indicates good balance.

In the multivariable regression models adjusting for age, BMI, herniation calcification, and migration grade, the perioperative advantages of PTED remained statistically significant. Specifically, PTED was independently associated with shorter operative time, reduced blood loss, and smaller incision length. The early postoperative difference in back pain VAS at 1 month also persisted after adjustment. Detailed results of the adjusted comparisons for key surgical parameters are presented in Table 4.Subgroup analyses revealed that the presence of herniation calcification or high BMI (>30 kg/m^2^) did not significantly modify the treatment effect of PTED vs. UBE on primary outcomes (interaction *P* > 0.05 for all).

**Table 4:** Comparison of surgical outcomes: unadjusted and adjusted analyses.

Outcome (Unit)	Unadjusted MD (95% CI)	*P* value	Adjusted MD (95% CI)	*P*
Operative Time (min)	−42.9 (−48.2, −37.6)	<0.001	−41.2 (−47.1, −35.3)	<0.001
Incision Length (mm)	−16.8 (−18.2, −15.4)	<0.001	−16.5 (−17.9, −15.1)	<0.001
Blood Loss (mL)	−49.7 (−53.3, −46.1)	<0.001	−48.6 (−52.9, −44.3)	<0.001
Hospital Stay (days)	−1.1 (−1.8, −0.4)	0.002	−1.0 (−1.7, −0.3)	0.005

MD, mean difference; CI, confidence interval. Adjusted MD derived from multivariable linear regression models controlling for age, BMI, herniation calcification, and migration grade.

### 3.4 Comparison of VAS and ODI before and after surgery

Both groups showed significant improvement in VAS and ODI scores at all postoperative time points compared to preoperatively (*P* < 0.05). At the early 1-month follow-up, the PTED group had a lower VAS score for back pain but a slightly higher score for leg pain and ODI compared to the UBE group. From 3 months postoperatively onward, no significant differences were found between the two groups in any scores (*P* > 0.05). Repeated-measures ANOVA indicated a different improvement trajectory for back pain VAS between the groups (interaction *P* = 0.024). (See Table 5 for details).

**Table 5:** Comparison of clinical efficacy scores between the two groups at different time points.

Score/time point	PTED group (*n* = 38)	UBE group (*n* = 43)	*t*	*P*
VAS for Back Pain				
Preoperative	7.6 ± 1.2	7.4 ± 1.1	0.810	0.421
1 Month Postop	2.8 ± 0.9*	3.5 ± 1.1*	−3.206	**0.002**
3 Months Postop	2.1 ± 0.7*	2.3 ± 0.8*	−1.249	0.215
6 Months Postop	1.5 ± 0.6*	1.7 ± 0.7*	−1.406	0.164
Final Follow-up (12M)	1.2 ± 0.5*	1.3 ± 0.6*	−0.869	0.387
VAS for Leg Pain				
Preoperative	8.1 ± 0.9	7.9 ± 1.0	1.005	0.318
1 Month Postop	2.2 ± 0.8*	1.9 ± 0.7*	2.001	**0.049**
3 Months Postop	1.5 ± 0.6*	1.4 ± 0.5*	0.851	0.398
6 Months Postop	1.1 ± 0.5*	1.0 ± 0.4*	1.072	0.287
Final Follow-up (12M)	0.9 ± 0.4*	0.8 ± 0.4*	1.220	0.226
ODI (%, x¯ ± s)				
Preoperative	78.5 ± 6.3	77.2 ± 7.0	0.910	0.366
1 Month Postop	25.4 ± 5.8*	28.6 ± 6.5*	−2.487	**0.015**
3 Months Postop	18.2 ± 4.1*	19.7 ± 4.9*	−1.561	0.122
6 Months Postop	12.1 ± 3.5*	13.0 ± 3.8*	−1.163	0.248
Final Follow-up (12M)	9.3 ± 2.8*	9.8 ± 3.2*	−0.765	0.446

Data are presented as mean ± standard deviation (x̅ ± s). Between-group comparisons at each time point were performed using independent samples *t*-test. The repeated-measures ANOVA revealed a significant main effect of time for all scores (VAS-Back: *F* = 1,526.347, *P* < 0.001; VAS-Leg: *F* = 2,891.438, *P* < 0.001; ODI: *F* = 936.167, *P* < 0.001). A significant time × group interaction was found only for VAS-Back (*F* = 3.224, *P* = 0.024), indicating different improvement trajectories between groups for back pain. No significant main effect of group was observed for any score (all *P* > 0.05). Greenhouse-Geisser correction was applied when the assumption of sphericity was violated. A two-sided *P* value of <0.05 was considered statistically significant and is highlighted in bold. VAS, visual analogue scale; ODI, Oswestry disability index.

Bold numbers indicate that the two groups have statistical significance.

* There was a statistically significant difference compared with preoperative.

### 3.5 Comparison of complication rates between the two groups

No severe complications such as dural tear, nerve root injury, surgical site infection, or symptomatic hematoma occurred in either group during the perioperative period. Details of surgery-related events are summarized in Table 6.

**Table 6:** Summary of surgery-related complications.

Complication	PTED Group (*n* = 38)	UBE Group (*n* = 43)
Intraoperative		
Severe pain requiring supplemental analgesia	1 (2.6%)	—
Early Postoperative (≤30 days)		
Transient neurological deficit	1 (2.6%)^a^	—
Peritoneal effusion	—	1 (2.3%)^b^
Total Patients with Complications	2 (5.3%)	1 (2.3%)

—Indicates that this complication did not occur.

^a^ Resolved completely within one week with observation and neurotrophic medication.

^b^ Attributed to irrigation fluid, managed conservatively with diuretics and resolved within 5 days.

### 3.6 Postoperative imaging evaluation

Table 7 presents the postoperative imaging outcomes for both surgical techniques. Regarding the foraminal area, the PTED group measured (98.5 ± 12.3) mm^2^, and the UBE group measured (96.8 ± 11.7) mm^2^, with no statistically significant difference between the groups (t = 0.674, *P* = 0.312). For the facet joint resection ratio, the PTED group was (8.2 ± 2.1)%, significantly lower than the UBE group's (15.7 ± 3.8)% (*t* = 11.24, *P* < 0.001), indicating that the PTED technique better preserves the facet joint structure.

**Table 7:** Postoperative imaging outcomes.

Outcome Measure	PTED Group (*n* = 38)	UBE Group (*n* = 43)	*t*	*P*
Foraminal Area (mm^2^)	98.5 ± 12.3	96.8 ± 11.7	0.674	0.312
Facet Resection Ratio (%)	8.2 ± 2.1	15.7 ± 3.8	11.24	<0.001

### 3.7 Typical cases

**Figure 2:**
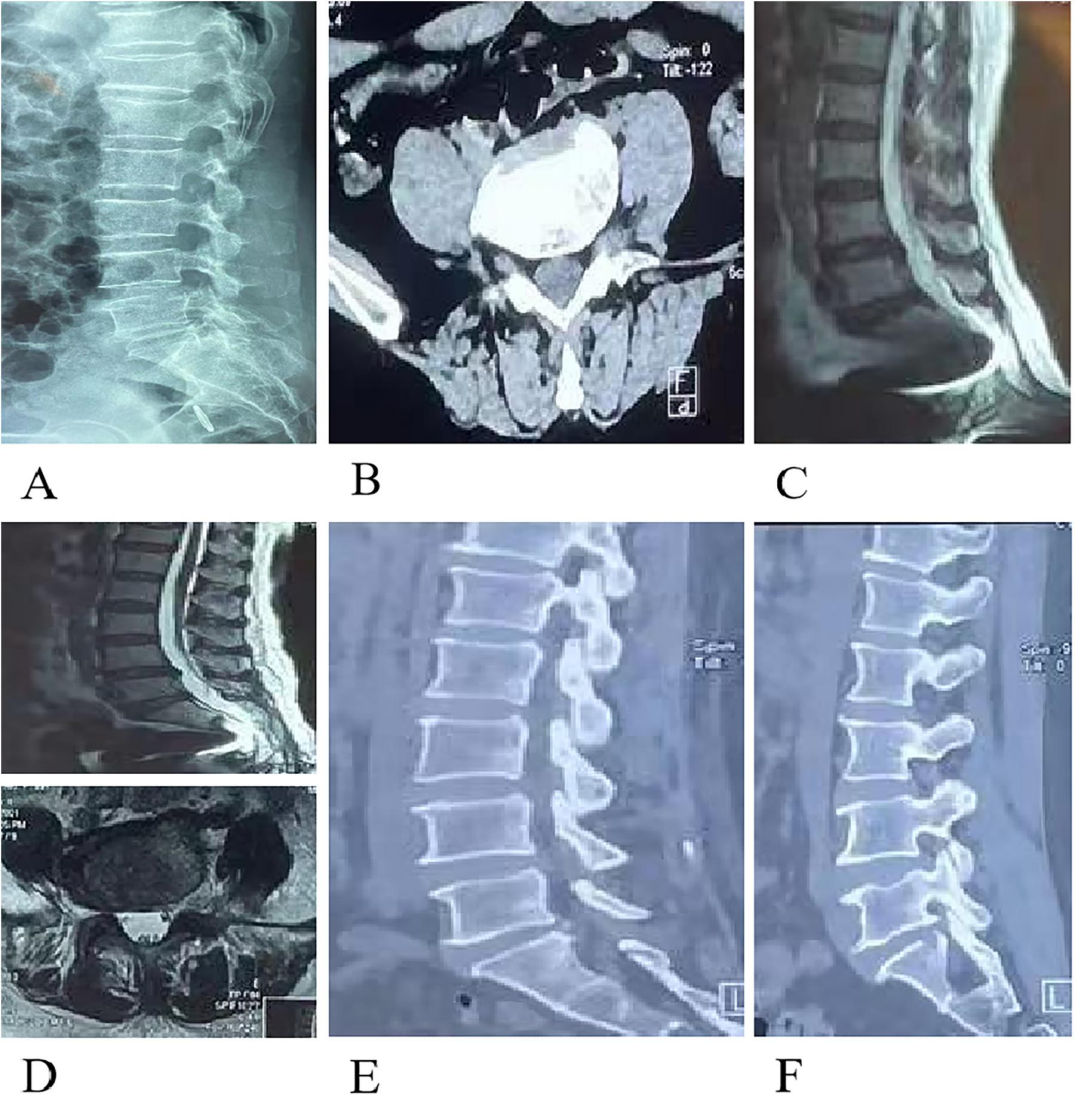
Preoperative and postoperative imaging in a patient with lumbar disc herniation treated with UBE. **(A)** Preoperative lateral lumbar radiograph indicates lumbar disc herniation at the L5-S1 level. **(B,C)** Preoperative lumbar CT and MRI show distal disc herniation at the L5-S1 level. **(D–F)** Lumbar CT and MRI performed 3 days postoperatively reveal partial absence of the lamina, with no significant abnormalities detected.

A 59-year-old female patient was admitted due to “numbness and pain in the left lower extremity.” See Figure 2 for details. (A) Preoperative lateral lumbar radiograph indicates lumbar disc herniation at the L5-S1 level. (B, C) Preoperative lumbar CT and MRI show distal disc herniation at the L5-S1 level. (D–F) Lumbar CT and MRI performed 3 days postoperatively reveal partial absence of the lamina, with no significant abnormalities detected.

## 4 Discussion

FLLDH, due to its unique anatomical location with direct compression on the exiting nerve root, often leads to severe radicular leg pain and neurological dysfunction (13). While traditional open decompression can be effective, it is frequently associated with extensive soft-tissue dissection and bony destruction, which may result in postoperative lumbar instability and chronic low back pain (14). With the rapid advancement of minimally invasive spinal techniques, PTED and UBE have become important minimally invasive options for treating FLLDH (15–18). This retrospective comparative study aimed to systematically evaluate the differences between these two techniques in terms of early clinical efficacy, perioperative parameters, and safety profiles, thereby providing evidence-based guidance for surgical decision-making. Our study incorporated propensity score matching and multivariable regression to control for key potential confounders, including herniation morphology and obesity—factors acknowledged to influence surgical outcomes. The persistence of significant differences in perioperative metrics after these adjustments strengthens the conclusion that the advantages of PTED in operative efficiency and reduced tissue trauma are robust and less likely attributable to baseline imbalances.

Our results demonstrate that both PTED and UBE significantly improved low back and leg pain symptoms as well as functional status in patients with FLLDH. Postoperative VAS and ODI scores showed marked reduction compared with preoperative values, with sustained improvement over time. By the 12-month follow-up, no statistically significant differences were observed between the two groups in any clinical scores, indicating comparable mid-term clinical outcomes. These findings are consistent with the study by Zhang et al. (19), which also concluded that both PTED and UBE are effective minimally invasive approaches for FLLDH, achieving satisfactory neural decompression and symptom relief. Although PTED demonstrated statistically lower back pain VAS and UBE showed lower leg pain VAS and ODI at the 1-month follow-up, the magnitude of these differences (0.3–0.7 points on VAS, 3.2% on ODI) did not meet the established Minimal Clinically Important Difference (MCID) thresholds for lumbar spine outcomes (20). This suggests that while the recovery trajectories may differ slightly in speed, the early clinical benefit perceived by patients is comparable between the two techniques.

Regarding perioperative indicators, the PTED group exhibited significant advantages over the UBE group in operative time, incision length, intraoperative blood loss, and length of hospital stay, suggesting that PTED is a more minimally invasive and efficient procedure (21). The UBE technique, requiring the establishment of two portals and more extensive soft-tissue dissection along with a higher degree of facetectomy (as evidenced by a significantly greater facet joint resection ratio in our imaging analysis), was associated with greater surgical trauma and blood loss. Our imaging analysis revealed that while both techniques provided comparable foraminal decompression, PTED was associated with a significantly lower facet joint resection ratio. This more conservative bony removal in PTED may better preserve segmental stability. Conversely, the greater facet resection in UBE, while creating a more spacious working corridor and aiding in visualization, likely contributes to the slightly higher early back pain VAS scores observed in this group. These findings are consistent with previous reports (22, 23). Similarly, Zhang et al. (19) reported longer operative time, larger incisions, and greater blood loss in the UBE group, further supporting the superior minimally invasive profile of PTED.

It is noteworthy, however, that despite its advantages in minimal invasiveness, PTED has a steep learning curve and may cause significant intraoperative pain under local anesthesia, especially when dealing with large, calcified, or mixed-type herniations (21, 24). In our study, one patient in the PTED group experienced intolerable pain requiring additional analgesia, highlighting the importance of patient tolerance and surgeon expertise. In contrast, UBE is performed under general anesthesia, eliminating intraoperative pain, and its operative logic resembles that of open surgery, offering greater instrument flexibility and a relatively gentler learning curve. UBE is particularly advantageous for complex cases or high-level FLLDH and is less restricted by anatomical obstacles such as a high iliac crest or hypertrophic transverse processes, allowing more direct visualization and decompression of the nerve root (25).

Concerning complications, a systematic overview is provided in Table 6. Both techniques demonstrated a favorable safety profile, with no instances of serious nerve injury, dural tear, or surgical site infection. The complications observed were technique-specific. In the PTED group, complications included one case (2.6%) of intraoperative pain requiring supplemental analgesia, highlighting a challenge of procedures under local anesthesia, and one case (2.6%) of transient postoperative dysesthesia that resolved completely within a week. In the UBE group, one patient (2.3%) developed a self-limiting peritoneal effusion, likely related to irrigation fluid dynamics during biportal surgery, underscoring the importance of careful fluid management. The overall low complication rates in this series reflect the procedures being performed by a specialized, high-volume surgical team proficient in both techniques, which may affect the generalizability of these perioperative outcomes to different settings.

Beyond these perioperative events, the choice of technique may also influence longer-term outcomes. Although PTED is less invasive, its limited working space and relatively conservative discectomy may be associated with a higher risk of recurrence, reported in the literature to be approximately 0%–6.9% (16, 26). Therefore, for younger patients, those with large herniations, or individuals with high physical demands, UBE may provide more thorough decompression and a potentially lower recurrence rate (27, 28).

This comparative study demonstrates that both PTED and UBE are effective minimally invasive techniques for single-level FLLDH, yielding comparable and excellent mid-term clinical improvement. While early (1-month) statistical differences in pain scores were observed, they did not reach the threshold of minimal clinically important difference (MCID), supporting the overall clinical equivalence of the two techniques. PTED demonstrated superior perioperative efficiency, including shorter operative time, smaller incisions, and less blood loss, indicating reduced surgical trauma. UBE provided superior endoscopic visualization under general anesthesia, which may enhance procedural control and safety in complex anatomical scenarios. When performed by experienced surgeons, the selection between these techniques may be individualized based on patient-specific factors, herniation morphology, and anatomical considerations. It should be noted that these findings are derived from a retrospective cohort in a specialized center with 12-month follow-up. Our findings warrant further validation in broader clinical practice. Further prospective, multicenter studies with longer follow-up are warranted to confirm long-term outcomes and refine evidence-based selection criteria.

This study has several inherent limitations. First, the single-center design with all procedures performed by a fixed team of senior spinal surgeons, while strength in terms of internal consistency and control over technical variables, limits the generalizability of our findings. The outcomes, particularly the favorable perioperative metrics and low complication rates, may reflect a high level of subspecialty expertise and volume. Consequently, they may not be directly replicable in community practice settings, during a surgeon's initial learning curve, or in centers with different patient populations and resources. Our results thus represent the potential of each technique under optimized conditions and should be validated in broader, multi-center settings. Second, the sample size, although sufficient for initial comparative assessment, remains relatively modest, which may limit the statistical power to detect subtle differences in outcomes or rare complications. Third, the follow-up duration was restricted to 12 months, which is adequate for evaluating early and mid-term efficacy but insufficient for assessing long-term outcomes such as symptom recurrence, adjacent segment degeneration, and the impact on lumbar spinal stability. Fourth, all surgical procedures were performed by a highly experienced and specialized spinal team proficient in both techniques, which may not reflect outcomes during the learning curve or in less specialized centers. Finally, the assessment of surgical trauma and recovery relied on standard perioperative metrics; future studies could incorporate more objective measures such as paraspinal muscle imaging (MRI) or serum inflammatory markers to quantify tissue injury and the systemic response.Fifth, although we employed propensity score matching and multivariable adjustment to mitigate confounding, our retrospective observational design remains susceptible to residual confounding from unmeasured variables, such as subtle anatomical variations, surgeon's intraoperative decision-making, or patient rehabilitation adherence. These factors could still influence outcomes independently of the surgical technique itself. Sixth, our assessment of clinical relevance relied on general MCID thresholds from the literature. Patient-perceived improvement can be individual, and future studies might incorporate patient-specific anchor questions (e.g., the Global Rating of Change scale) to further personalize the assessment of clinical importance. Seventh, as a retrospective study, the range of outcome measures was necessarily confined to those routinely documented in our clinical practice. While we captured core metrics such as pain and disability scores, standardized and detailed assessments of neurological recovery (e.g., motor strength grading, sensory mapping) and specific patient-reported outcomes (e.g., satisfaction scores, time to return to work or daily activities) were not systematically recorded for all patients. This limits our ability to fully evaluate the comprehensive functional benefits and patient-perceived success of these minimally invasive techniques. Future prospective studies should prioritize the inclusion of these patient-centered and functional recovery metrics.

## 5 Conclusions

This comparative study suggests that PTED and UBE yield comparable mid-term clinical improvement in patients with single-level FLLDH. PTED demonstrates advantages in perioperative metrics such as operative time, incision length, and blood loss, indicating reduced surgical trauma. UBE offers superior endoscopic visualization and is performed under general anesthesia, which may enhance procedural comfort and safety in complex anatomical scenarios. The selection between these techniques may be guided by patient-specific factors, anatomical challenges, and surgeon expertise, with the understanding that findings are derived from a retrospective cohort with 12-month follow-up. Further prospective studies with longer follow-up are warranted to confirm long-term outcomes and refine clinical decision-making.

## Data Availability

The raw data supporting the conclusions of this article will be made available by the authors, without undue reservation.
